# Dystonia, facial dysmorphism, intellectual disability and breast cancer associated with a chromosome 13q34 duplication and overexpression of *TFDP1*: case report

**DOI:** 10.1186/1471-2350-14-70

**Published:** 2013-07-13

**Authors:** Mariana Moscovich, Mark S LeDoux, Jianfeng Xiao, Garrett L Rampon, Satya R Vemula, Ramon L Rodriguez, Kelly D Foote, Michael S Okun

**Affiliations:** 1 Center for Movement Disorders and Neurorestoration, Department of Neurology, University of Florida College of Medicine, 100 S Newell Drive, Room L3-101, Gainesville, FL 32610, USA; 2 Department of Neurology, University of Tennessee Health Science Center, 855 Monroe Avenue, Suite 415 Link Building, Memphis, Tennessee 38163, USA; 3 Center for Movement Disorders & Neurorestoration, Department of Neurosurgery, University of Florida, McKnight Brain Institute, 100 S Newell Drive, Room L2-100, Gainesville, FL 32610, USA

**Keywords:** Dystonia, Chromosome 13q34, Duplication, *TFDP1*, Breast cancer, G_1_-S Checkpoint pathway

## Abstract

**Background:**

Dystonia is a movement disorder characterized by involuntary sustained muscle contractions causing twisting and repetitive movements or abnormal postures. Some cases of primary and neurodegenerative dystonia have been associated with mutations in individual genes critical to the G_1_-S checkpoint pathway (*THAP1*, *ATM*, *CIZ1* and *TAF1*). Secondary dystonia is also a relatively common clinical sign in many neurogenetic disorders. However, the contribution of structural variation in the genome to the etiopathogenesis of dystonia remains largely unexplored.

**Case presentation:**

Cytogenetic analyses with the Affymetrix Genome-Wide Human SNP Array 6.0 identified a chromosome 13q34 duplication in a 36 year-old female with global developmental delay, facial dysmorphism, tall stature, breast cancer and dystonia, and her neurologically-normal father. Dystonia improved with bilateral globus pallidus interna (GPi) deep brain stimulation (DBS). Genomic breakpoint analysis, quantitative PCR (qPCR) and leukocyte gene expression were used to characterize the structural variant. The 218,345 bp duplication was found to include *ADPRHL1*, *DCUN1D2*, and *TMCO3*, and a 69 bp fragment from a long terminal repeat (LTR) located within Intron 3 of *TFDP1*. The 3*'* breakpoint was located within Exon 1 of a *TFDP1* long non-coding RNA (NR_026580.1). In the affected subject and her father, gene expression was higher for all three genes located within the duplication. However, in comparison to her father, mother and neurologically-normal controls, the affected subject also showed marked overexpression (2×) of the transcription factor *TFDP1* (NM_007111.4). Whole-exome sequencing identified an *SGCE* variant (c.1295G > A, p.Ser432His) that could possibly have contributed to the development of dystonia in the proband. No pathogenic mutations were identified in *BRCA1* or *BRCA2*.

**Conclusion:**

Overexpression of *TFDP1* has been associated with breast cancer and may also be linked to the tall stature, dysmorphism and dystonia seen in our patient.

## Background

Chromosome 13q34 amplifications have been reported in lung cancer [1], small bowel adenocarcinoma [2], hepatocellular carcinoma [3], squamous cell carcinoma of the esophagus [4], and breast cancer [5,6]. Overexpression of genes in this region, particularly *TFDP1*, may be causally associated with the development or progression of these malignancies [5,6]. *TFDP1* encodes the transcription factor DP-1. DP-1 forms heterodimers with E2F family members to enhance their DNA-binding and promote transcription of E2F target genes. The E2F/DP-1 complex plays a critical role in the G_1_-S cell-cycle transition [7].

Numerous stimuli, including DNA damage, TGFβ, replicative senescence, and contact inhibition, exert control over the G_1_-S checkpoint pathway [8,9]. Defects in this pathway have been associated with developmental defects, neurodegenerative disorders, cancer and dystonia [10-12]. In particular, five genes linked to dystonia appear to be involved upstream or downstream of DP-1 in the G_1_-S checkpoint pathway (*ATM*, *CIZ1*, *TOR1A*, *THAP1* and *TAF1*). Autosomal recessive mutations of *ATM* increase risk for malignancies and typically present as ataxia telangiectasia. However, some patients present with isolated dystonia [11]. Moreover, female carriers are at higher risk for breast cancer [13]. The encoded protein, ataxia telangiectasia mutated (ATM), activates p53 by phosphorylating the ubiquitin E3 ligase MDM2 [14]. Reduced neuronal expression of *TAF1* is linked to a form of dystonia-parkinsonism known as Lubag or DYT3 [15]. TAF1 mediates degradation of p53 via phosphorylation at Thr-55 [16]. CIZ1 interacts with p21Cip1 [17]. TorsinA, the protein encoded by *TOR1A*, interferes with TGFβ signaling pathway [18]. Finally, the S-phase gene *RRM1* is a direct transcriptional target of THAP1 [19].

Herein, we report a unique patient with a chromosome 13q34 duplication, dystonia, dysmorphism, intellectual disability, psychosis, tall stature, and breast cancer. Gene expression studies suggest that one or more of these phenotypic features may be due to overexpression of *TFDP1*.

## Case presentation

A 36 year-old right-handed Hispanic woman presented for evaluation of stiffness, painful muscle spasms, difficulty walking, falling, and abnormal involuntary hyperkinetic movements that had progressed over three years. She also reported decreased motivation, speech articulation problems, and difficulty with all activities of daily living as well as with handwriting. The chronology of proband’s signs and symptoms is outlined in Table 1.

**Table 1:** Chronology of proband’s signs and symptoms

	
1976	Born by cesarean section at full term.
1976-1986	Did not sit until after 6 months of age and did not walk until 18 months of age.
	Enrolled in special education classes in the first grade.
	Developmental delay, Marfanoid features, dysmorphic features, strabismus, mitral valve prolapse were described by pediatrician.
2003-2007	Appearance of psychiatric symptoms including psychosis treated with valproic acid, risperidone and buproprion.
2008	Movement disorder becomes manifest (choreoathetotic and dystonic movements in the left arm along with blepharospasm) and is treated with levetiracetam, carbamazepine, oxcarbazepine, lorazepam, biperiden, haloperidol, aripiprazole, topiramate, chlorpromazine, carbidopa/levodopa, baclofen, amantadine, trihexyphenidyl, botulinum toxin injections, and tetrabenazine.
2009	Despite medical treatment and discontinuation of dopamine blocking drugs, neurological condition progressively worsens with hyperkinetic movements appearing in the right arm and trunk.
2010	Mucinous carcinoma of the right breast diagnosed and treated with a modified radical mastectomy with skin preservation and immediate reconstruction with breast prosthesis.
	Histology report shows no residual tumor at tissue margins and lymph nodes free of tumor.
	Post-operative treatment with tamoxifen.
	Worsening of the dystonia with appearance of truncal dystonia including painful retrocollis.
	Difficulty staying upright for even short periods.
	Weight loss of 25 pounds.
2011	Implantation of bilateral globus pallidus internus electrodes for deep brain stimulation.
	Marked improvement of dystonia 4 months after surgery.
	Subject able to feed herself, sit upright and walk.
	Significant improvements in neck pain.
	Revision of left and right pulse generators, and replacement of left extension cable after lead fracture due to twiddler syndrome (obsessively moving the generators in the chest leading to device fracture).
2013	Facial dystonia markedly improved. Sustained control of dystonia with deep brain stimulation.

She was born by cesarean section and was noted to be hypoxemic but was delivered at full term. She was the fourth child in her family, and none of the other children had neurological disorders or cancer related illnesses. Her motor and cognitive development was delayed. She did not sit until after 6 months of age and did not walk until 18 months of age, and was enrolled in special education classes in the first grade.

Because of her tall stature at 7 years of age, a diagnosis of Marfan syndrome was suggested by her physicians but never confirmed with molecular genetic testing. She completed a total of 6 years of schooling. At 30 years of age, she developed a psychiatric disturbance with psychotic features. She became obsessed with popular rock and roll bands and experienced visual and auditory hallucinations. She was briefly hospitalized for psychosis, and was treated successfully for three years with risperidone (1 mg three times daily), valproic acid and buproprion. However, she then began to manifest what was described as choreoathetotic and dystonic movements in her left arm along with blepharospasm. She was treated with levetiracetam, carbamazepine, oxcarbamazepine, clonazepam, biperidone, haldoperidol, aripiprazole, topiramate, baclofen, carbidopa/levodopa, chlorpromazine, amantadine, trihexyphenidyl, and botulinum toxin injections. These medical treatments were only mildly beneficial, and her neurological condition progressively worsened with the appearance of hyperkinetic movements affecting her right arm and trunk.

At age 35, she was diagnosed with breast cancer during a routine physical examination. She underwent a radical right mastectomy with preservation of the areola and nipple. Serum prolactin levels were not obtained. She was treated with tamoxifen, post-operatively, and has shown no evidence of local recurrence or metastasis of her breast cancer. Pathology revealed an encapsulated mucinous carcinoma, grade I. The tumor cells inside mucous lakes expressed estrogen and progesterone receptors and did not over-express Her2/neu.

Her hyperkinetic movement disorder showed an initial modest response to tetrabenazine. However, dystonia gradually worsened with painful retrocollis and truncal dystonia. Over a period of 6 months, she lost 25 pounds and became profoundly debilitated by dystonia, spending most of her waking day lying face down.

Brain magnetic resonance image (MRI) was unremarkable and routine laboratory testing including a comprehensive metabolic profile was normal. A 24-hour urine copper for Wilson’s disease was also normal. A 99mTc-HMPAO single photon emission computed tomography (SPECT) showed asymmetric and decreased radiotracer uptake in basal ganglia regions including the caudate, putamen, and globus pallidus. Molecular cytogenetic analysis exposed a chromosome 13q34 duplication.

**Figure 1:**
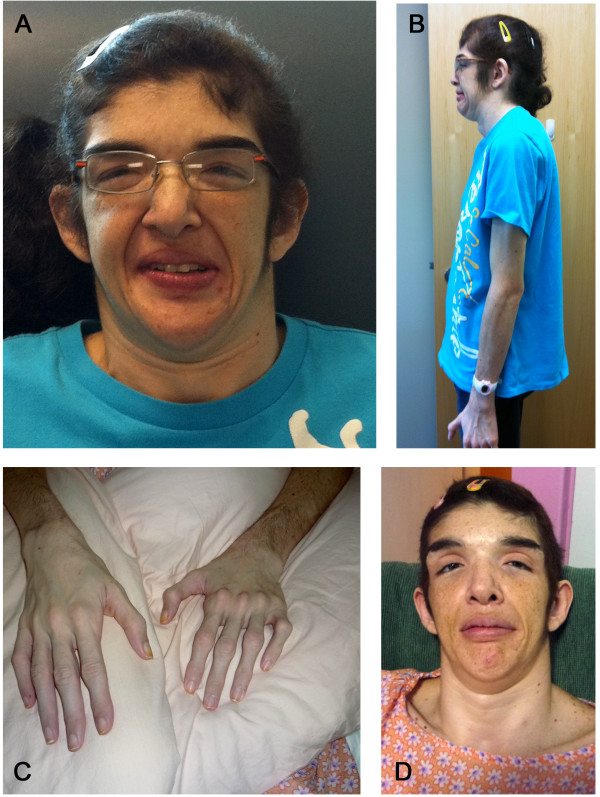
**Frontal (A) and lateral (B) photographs 4 months after GPi DBS show the presence of mild facial dystonia and dysmorphic features. C)** Arachnodactyl and hirsutism. **D)** Virtual resolution of facial dystonia 2 yrs after GPi DBS.

At age 36, general physical examination revealed facial dysmorphism with hypertelorism, down slanting palpebral fissures, mild ptosis and large low-set ears with mild posterior rotation (Figure 1). Other clinical features included hirsutism, low anterior hairline, micrognathia, brevicollis, scoliosis, arachnodactyly, tall stature (182.9 cm), and a weight of 62.3 kg (body mass index = 18.6). On neurological examination, she was moderately inattentive. No primitive reflexes were noted. She scored a 19/30 on the Montreal Cognitive Assessment (MoCA). MoCA scores of 26 and higher are considered normal. Although cranial nerves were all normal, speech was mildly dysarthric, and facial dystonia was overt. Muscle strength testing was normal. There was no focal atrophy or fasciculations. There was no obvious upper extremity ataxia although testing of coordination was limited by the presence of dystonia. Gait was broad-based and ataxic, and she required assistance to walk. Walking was also compromised by dystonic posturing of her trunk and arms. Sensory testing was normal. Cervical dystonia was manifest as severe retrocollis. Truncal and bilateral arm dystonia had a clear action component and worsened during movement, particularly walking. No contractures were present in the arms or legs. A score of 37 was recorded for the pre-operative Unified Dystonia Rating Scale (UDRS, maximum score is 112).

The proband underwent bilateral globus pallidus internus (GPi) deep brain stimulation (DBS). She gradually improved and after 4 months her facial dystonia was markedly better and she became able to feed herself. She could sit upright and tolerated sitting for progressively longer periods of time. Both subjective and objective improvements in postural stability, gait, and retrocollis were noted. Neck pain and upper limb dystonia were significantly improved. The 4-month post-operative score on the UDRS was 23. However, shortly after this period of clinical improvement, she experienced hardware malfunction due to lead wire fractures caused by her twiddling the implantable pulse generators (IPGs). The lead connectors were replaced and the IPGs tacked down to prevent twiddling, and her dystonia improved once again. Her outpatient clinical course has been compromised by dysphagia and hospitalizations for treatment of aspiration pneumonia which ultimately required placement of a percutaneous endoscopic gastrostomy (PEG) tube.

The proband’s parents and two brothers were neurologically normal and showed no dysmorphic features. Her mother and father were 167.6 and 170.2 cm tall, respectively. There was no history of dystonia or breast cancer in any first or second degree relative.

All human studies were performed in accordance with institutional review board guidelines and the Helsinki Declaration. Written informed consent for genetic studies and publication of clinical data was obtained from the patient, her parents and neurologically-normal controls. All genetic analyses were approved by the University of Tennessee Health Science Center Institutional Review Board (#01-07346-XP). The proband and her parents were examined at the Center for Movement Disorders and Neurorestoration, Department of Neurology, University of Florida, and were part of the IRB approved INFORM clinical database. The proband’s siblings live outside the United States and were unable to complete a study visit at the University of Florida. However, the siblings were interviewed via Skype (http://www.skype.com). Recruitment of neurologically-normal controls has been described previously [20].

### Identification of the duplication

Clinical cytogenetic analyses were performed by Quest Diagnostics (Madison, NJ, USA) on DNA derived from whole blood using the Affymetrix Genome-Wide Human SNP Array 6.0. The Affymetrix 6.0 microarray contains over 1.8 million probes, including 900,000 copy number probes and 900,000 SNP probes. The average inter-probe distance is 700 base pairs. Thresholds for genome-wide screening were set at >200 kb for gains, >50 kb for losses and 10 Mb for segments of homozygosity. A ~225 kb gain of unclear clinical significance was detected in the proband at 13q34. Four genes were involved in the genomic alteration: *ADPRHL1*, *DCUN1D2*, *TMCO3* and *TFDP1*. Follow-up analyses of the parents using the same array indicated that the variant was of paternal origin. The classic *TOR1A* ΔGAG mutation was excluded with Sanger sequencing.

For genomic breakpoint analysis and confirmation of copy numbers, DNA was extracted from peripheral blood leucocytes using Roche’s DNA Isolation Kit for Mammalian Blood (Indianapolis, IN, USA). DNA quantity and quality were analyzed with a NanoDrop ND-1000 spectrophotometer (Wilmington, DE, USA) and agarose gel electrophoresis. Quantitative PCR (qPCR) primers and probes were designed via Roche’s Universal Probe Library to encompass the estimated boundary regions (Additional file 1). qPCR was performed using 20 ng of template DNA and 200 nM of each primer in a 10-μl reaction volume with the LightCycler™ 480 system and Universal Taqman® probes (Roche). The following cycling conditions were employed: 95°C for 5 min; 45 cycles at 95°C for 10 s, 60°C for 30 s, and 72°C for 12 s. Copy numbers were referenced to an endogenous control (*RPPH1*) and 9 neurologically-normal unrelated subjects. All assays were done in triplicate and means were used for comparisons. Long-range PCR was then performed using primers outside the boundary regions of the duplication (for example, Dup237_p13F and Dup025_p23R in Additional file 1). After agarose gel purification, PCR products were sequenced in the forward and reverse directions on an Applied Biosystems 3130XL Genetic Analyzer (Carlsbad, CA, USA).

Relative quantitative reverse-transcriptase PCR (QRT-PCR) was done to analyze the expression levels of genes located within or near the structural variant. In brief, Ambion’s LeukoLOCK™ Total RNA Isolation System and TRI Reagent® were used to isolate RNA from peripheral blood leukocytes. Leukocyte RNA was not available for the proband’s siblings. Reverse transcription was performed with Ambion's RETROscript™ kit using 500 ng total RNA as template along with random oligonucleotide primers. The reaction mixtures were incubated at 44°C for 1 hr and then at 92°C for 10 min. QRT-PCR was performed using the LightCycler^TM^ 480 system with gene specific primers (Additional file 1) and Universal Taqman® probes (Roche) for 4 genes involved in the duplication region (*ADPRHL1, DCUN1D2, TMCO3*, and *TFDP1*)*, TFDP1* long non-coding RNA (lncRNA), 1 gene near the duplication (*GRTP1*), and the endogenous control (*PPID*), which encodes 40 kDa peptidyl-prolyl cis-trans isomerase D (cyclophilin D). All assays were performed in triplicate and means were used for comparisons.

**Figure 2:**
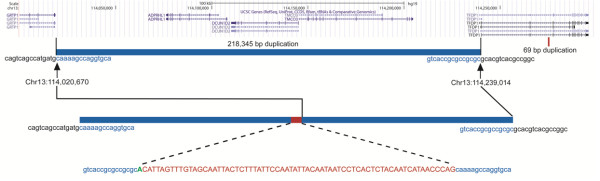
**Genomic rearrangement at 13q34.** The 218,345 bp duplication is shown beneath gene tracks adapted from the UCSC Genome Browser (GRCH37/hg19 Assembly). The nucleotide sequence for the 69 bp fragment, possibly duplicated from an LTR located in Intron 3 of *TFDP1*, is shown in upper case red lettering.

Identification of exact breakpoints was facilitated by mapping flanking SNPs and Affymetrix copy number (CN) probes for the 13q34 duplication in both the proband and her father (bolded were predicted by Quest Diagnostics to be within the gain): SNP_A-1803536 (Chr13:114022946), CN_634194 (Chr13:114023445)***,*** SNP_A-8304155 (Chr13:114023625), **CN_091810(Chr13:114025424)*, *CN_634195(Chr13:114027458)*, *SNP_A-2261043(Chr13:114030927)*, *SNP_A-8505553(Chr13:114249807), CN_091834(Chr13:114249942)**, CN_636300 (Chr13:114257699), and SNP_A-2105146(Chr13:114257910). With qPCR of genomic DNA we were able to confirm duplication of *ADPRHL1*, *DCUN1D2*, and *TMCO3*, and showed that *GRTP1* and *TFDP1* were, in fact, outside the duplicated segment (Figure 2 and Table 2). Fold changes of approximately 1.5× for *ADPRHL1*, *DCUN1D2* and *TMCO3* were consistent with a duplication involving one allele of each gene. Stepwise long-range PCR and Sanger sequencing allowed us to identify the 5′ and 3′ breakpoints (Chr13:114,020,670 and Chr13:114,239,014) in the affected subject and her father (Figure 2). The duplicated segments were separated by a 69 bp fragment. Nucleotide query with Basic Local Alignment Search Tool (BLAST) suggested that this 69 bp fragment (Chr13:114,275,816-884) was duplicated from Intron 3 of *TFDP1*, 36 kb 3′ to its location between the larger duplicated segment. A single deoxyadenosine (A) was detected 5′ to the smaller 69 bp fragment. The 5′ breakpoint (Chr13:114,020,670) was located 3′ to the end of *GRTP1* (Chr13:114,018,463) and the 3′ breakpoint (Chr13:114,239,014) was located 5′ to the start of *TFDP1* (NM_007111.4: Chr13:114,239,056), within Exon 1 of a *TFDP1* long non-coding RNA (NR_026580.1). The 69 bp fragment showed 100% sequence identity to a long terminal repeat (LTR) of the endogenous retrovirus family K (ERVK) located in Intron 3 of *TFDP1*. This LTR is 983 nt in length (Chr13:114,275,682-114,276,664). Collectively, the complex structural variant identified in our patient and her father can be denoted as *NC_000013.10:g.114,020,670_114,239,014dup218,345;g.114,239,014_114,239,015ins(A + 114,275,816_114,275,884).* This variant was also identified in one of the proband’s three brothers (Table 3). It has not been reported in the Database of Genomic Variants (http://projects.tcag.ca/variation/).

**Table 2:** Quantitative PCR of genomic DNA

**Primers**	**Location (13q34)**	**Normal controls**	**Subject fold change**		
	**NC_000013.10**	**(N = 9)**	**Patient**	**Father (carrier)**	**Mother**
Dup_018F/R	114018980-9123	1.00 ± 0.13	0.94	0.98	1.01
Dup_023F/R	114023386-3429	1.00 ± 0.15	1.47	1.51	0.99
Dup_025F/R	114025267-5677	1.00 ± 0.17	1.52	1.58	1.02
Dup_041F/R	114041257-1333	1.00 ± 0.12	1.57	1.49	0.98
Dup_228F/R	114228694-8793	1.00 ± 0.18	1.61	1.58	1.08
Dup_237F/R	114237214-7282	1.00 ± 0.09	1.53	1.55	1.02
Dup_241F/R	114241291-1364	1.00 ± 0.16	0.95	0.99	0.98
Dup_249F/R	114249685-9762	1.00 ± 0.10	1.10	1.04	1.01
Dup_257F/R	114257807-7900	1.00 ± 0.11	1.08	0.99	1.00

**Table 3:** Pedigree genotypes

**Family member**	**Age**	**Phenotype**	**13q34 Duplication**	***SGCE *Exon10**	***BRCA1*, rs799917**	***XRCC3*, rs861539**
					**NC_000017:**	**NC_000014:**
					**g.41244936G > A**	**g.104165753G > A**
Proband	37	Generalized dystonia, breast cancer, dysmorphism	Yes	c.1294A > C (homozygous), c.1295G > A (heterozygous)	heterozygous	heterozygous
Father	71	Normal	Yes	c.1294A > C (homozygous), c.1295G > A (heterozygous)	heterozygous	wild-type
Mother	70	Normal	No	c.1294A > C (homozygous)	heterozygous	heterozygous
Brother	47	Normal	No	c.1294A > C (homozygous)	heterozygous	wild-type
Brother	46	Normal	No	c.1294A > C (homozygous)	heterozygous	heterozygous
Brother	43	Normal	Yes	c.1294A > C (homozygous), c.1295G > A (heterozygous)	heterozygous	heterozygous

Relative gene expression in leukocytes is shown in Table 4. In the affected subject and her father, relative RNA expression was higher for all three genes located within the duplication region (*ADPRHL1, DCUN1D2* and *TMCO3*). However, in comparison to her mother and neurologically normal controls, the affected subject showed marked overexpression (>2×) of *ADPRHL1* and *TMCO3*. In contrast, gene expression in the father was approximately 0.5× above control values for *ADPRHL1*, *DCUN1D2* and *TMCO3*, compatible with the presence of three copies of these genes. Of particular note, *TFDP1* and *TFDP1* lncRNA showed strong leukocyte overexpression in the affected subject but not in her father or mother. For gene expression analyses, all assays were performed in triplicate and means were used for comparisons.

**Table 4:** Relative leukocyte gene expression

**Gene**	**Location of the gene**	**Normal controls**	**Subject fold change**		
		**(N = 9)**	**Patient**	**Father (carrier)**	**Mother**
*ADPRHL1* P1	Within duplication	1.00 ± 0.18	2.71	1.70	1.10
*ADPRHL1* P2		1.00 ± 0.25	2.26	1.70	1.10
*DCUN1D2* P1	Within duplication	1.00 ± 0.05	1.48	1.61	0.95
*DCUN1D2* P2		1.00 ± 0.19	1.62	1.46	0.96
*TMCO3* P1	Within duplication	1.00 ± 0.13	3.13	1.68	1.04
*TMCO3* P2		1.00 ± 0.06	2.69	1.59	1.21
*TFDP1* P1	3*'* to duplication	1.00 ± 0.20	1.95	1.05	1.25
TFDP1 P2		1.00 ± 0.16	2.04	0.97	1.18
*TFDP1* lncRNA P1	3*'* to duplication	1.00 ± 0.18	2.01	0.93	1.20
*TFDP1* lncRNA P2		1.00 ± 0.21	2.15	0.95	1.11
*GRTP1* P1	5*'* to duplication	1.00 ± 0.13	1.08	0.89	1.26
*GRTP1* P2		1.00 ± 0.10	0.98	0.85	1.28

### Exome sequencing and variant analysis

To identify other genetic factors that could cause or contribute to risk of developing breast cancer and/or dystonia, in-solution whole-exome capture and massively parallel sequencing using the Agilent SureSelect XT All Exon Kit 51 Mb (Santa Clara, CA, USA) was used to examine the proband’s exome. Three micrograms of genomic DNA from the subject was sheared to yield 100–450 bp fragments. Sheared DNA was then subjected to Illumina paired-end library preparation followed by enrichment for target sequences (Agilent SureSelect^XT^ Automated Target Enrichment for Illumina Paired-End Multiplexed Sequencing). Enriched DNA fragments were sequenced on Illumina’s HiSeq 2000 platform as paired-end 100 base reads (Otogenetics Co., Norcross, GA, USA) and reads were mapped to the human reference genome with NextGENe® (SoftGenetics, State College, PA, USA).

Over 99.7% of exons were covered at ≥2× and 97.6% of exons were covered at ≥20×. To maximize the chances of detecting a pathogenic variant, base changes occurring in two or more reads were classified as variants for downstream analyses. We focused on genes associated with either breast cancer or dystonia (Additional file 2). The pathogenicity of non-synonymous single amino acid substitutions was interrogated with six *in silico* tools: PolyPhen-2, MutationTaster, SIFT_new_, LRT_new_ (Likelihood Ratio Test), PhyloP_new_, and ClustalW2 [21-23]. Sequence variants in *SGCE*, *BRCA1*, and *XRCC3* were confirmed by Sanger sequencing in the forward and reverse directions. We identified a novel variant in *SGCE* (c.1295G > A, p.Ser432His) that is unlikely to be pathogenic (Table 3). Although predicted to be damaging by SIFT [24], p.Ser432His is poorly conserved [22] and classified as “benign” and “polymorphism” by Polyphen-2 [25] and MutationTaster [26], respectively. Whole exome sequencing also identified a *CIZ1* polymorphism that has not been associated with dystonia. Polymorphisms (dbSNP) were found in 16/25 breast cancer associated genes (Additional file 2). Two of these (*BRCA1*, rs799917; and *XRCC3*, rs861539) have shown potential but inconclusive associations with breast cancer [27,28].

### Discussion

Dystonia is a neurological disorder characterized by involuntary sustained muscle contractions producing abnormal postures, twisting and repetitive abnormal movements, and sometimes painful muscle contractions. During dystonic movements, agonist and antagonist muscles co-contract in abnormal patterns. Dystonia is typically classified into primary, secondary, heredodegenerative diseases with dystonia, and dystonia-plus etiological categories [29]. Mutations in *TOR1A*, *THAP1*, *CIZ1, ANO3, GNAL* and *TUBB4A* have been associated with some cases of primary dystonia [12,20,23,30-33]. However, the genetic etiologies for most patients with primary dystonia remain unknown. The dystonia-plus category includes the myoclonus-dystonia syndrome (MDS) secondary to *SGCE* mutations. Nearly all affected individuals have myoclonus and 50 to 65% manifest dystonia, typically in the neck and arms. Depression and anxiety are important non-motor features of MDS. Although our proband did harbor a potentially pathogenic *SGCE* missense variant, other carriers in the pedigree were asymptomatic. Moreover, the anatomical distribution and onset age of the proband’s dystonia were atypical for MDS.

Secondary dystonia is caused by exogenous factors such as medications, toxins, or brain damage that may result from head trauma and stroke. Drugs that block dopamine receptors, such as neuroleptics and antiemetics, can cause acute dystonic reactions or late-onset, persistent “tardive” dystonia, after months or years of treatment [34]. Tardive movement disorders are usually hyperkinetic and include classic tardive oral-buccal-lingual dyskinesias and tardive chorea, dystonia, tics, tremor and akathisia. The differentiation of tardive from primary dystonia can be difficult and categorization remains clinical as it was for our patient. In this regard, it is quite uncommon for patients with tardive dystonia to progressively worsen months after discontinuation of drugs that block dopamine receptors. Although the genetic susceptibility for developing tardive movement disorders has been poorly characterized, sequence variants in genes associated with primary dystonia could be risk factors [35,36]. For instance, a novel missense mutation in *TOR1A* (c.613 T > A) has been linked to adult-onset lower facial and masticatory, possibly tardive, dystonia [35].

It is now well-established that GPi DBS exerts beneficial effects on many dystonia subtypes [34-41]. In general, current experience with DBS suggests that primary dystonias respond better than most secondary dystonias [42]. However, many reports indicate that tardive dystonia can show significant improvements with DBS [42].

The complex structural variant identified in our patient is of uncertain clinical significance since the three duplicated genes have not been associated with dystonia, intellectual disability or dysmorphism in human populations. Moreover, two clinically unaffected family members harbored the same variant. *ADPRHL1* encodes an ADP-ribosyltransferase that transfers ADP-ribose from NAD + to target proteins. *DCUN1D2* encodes a protein that may contribute to neddylation of cullin components of SCF-type E3 ubiquitin ligase complexes. Neddylation is the process by which *NEDD8* is conjugated to target proteins. *NEDD8* is an ubiquitin-like modifier of protein function. Finally, *TMCO3* encodes a transmembrane protein that probably functions as a Na^+^/H^+^ antiporter. Chromosome 13q34 has, however, been linked to cancer, and, in Chinese, harbors a quantitative trait locus for physical height [43].

Pathogenic structural variants can be inherited from normal parents [44-47]. Penetrance values have been calculated for some of the more common structural variants and, for example, can range from 10.4% for 15q11.2 deletions to 62.4% for distal 16p11.2 deletions [47]. Some structural variants may be associated with several phenotypes and penetrance is phenotype specific. Ultimately, other genetic and environmental factors contribute to penetrance and associated phenotypes. In the case of our proband, neuroleptic exposure may have increased her risk for breast cancer [48]. However, whole-exome sequencing did not expose pathogenic non-structural variants in genes associated with either breast cancer or dystonia.

In our patient with dystonia and dysmorphism, it is possible that the presence of the breakpoint within the upstream promoter region of *TFDP1* (NM_007111.4) is responsible for altered expression of this transcription factor. The breakpoint could abrogate the effects of 5′ repressor regions on *TFDP1* expression. Over-expression of the *TFDP1* lncRNA could have also contributed to this effect since some lncRNAs have been shown to play important roles in transcriptional regulation [49]. In theory, the consequences of the breakpoint on *TFDP1* expression could be gender dependent. In this regard, *TFDP1* is part of the estrogen receptor ERα regulatory network [50]. Epistasis and epigenetics (DNA methylation or histone deacetylation) are additional mechanistic considerations [51] that could explain why the father and brother are asymptomatic carriers of the structural variant while the proband manifests dystonia due to overexpression of *TFDP1*. Conversely, *TFDP1* may have played no causal role in our patient’s dystonia and other clinical manifestations.

## Conclusions

This is the first report to describe a potential connection between *TFDP1* and dystonia. Moreover, our analyses suggest that a *TFDP1* lncRNA (NR_026580.1) could play a role in regulating *TFDP1* expression. Our genetic findings are compatible with numerous previous studies which have consistently identified a link between chromosome 13q34 and cancer. Further studies in large case–control cohorts will be needed to confirm possible associations between chromosome 13q34 and dystonia.

### Consent

Written informed permission for use and disclosure of this families protected health information for research purposes and informed consent was obtained from the patient for publication of this case report and any accompanying images. Copies of the consents are available for review by the Editor-in-Chief of this journal.

## Competing interests

The authors declare that they have no competing interests.

## Authors’ contributions

All authors reviewed the manuscript critically for its content, revised and edited it, and approved the final version. Additionally, specific author contributions are as follows: JX, GLR, SRV and MSL performed the genetic analyses; MM, RLR, KDF and MSO treated the patient and collected clinical data; MSL and MSO conceptualized, designed and coordinated the study; and MM, MSO and MSL generated the first draft of the manuscript. All authors read and approved the final manuscript.

## Pre-publication history

The pre-publication history for this paper can be accessed here:

http://www.biomedcentral.com/1471-2350/14/70/prepub

## Supplementary Material

Additional file 1 Primers used for PCR, QRT-PCR, qPCR, and Sanger sequencing. Click here for file

Additional file 2 Major genes associated with breast cancer. Click here for file
