# Supplementary material for: Dystonia, facial dysmorphism, intellectual disability and breast cancer associated with a chromosome 13q34 duplication and overexpression of TFDP1: case report
Source: BMC Med Genet. 2013 Jul 13;14:70. doi: 10.1186/1471-2350-14-70 (PMC3722009; doi:10.1186/1471-2350-14-70)
Supplement: Additional file 1 — Primers used for PCR, QRT-PCR, qPCR, and Sanger sequencing. [file 1471-2350-14-70-S1.docx]

**Primers used for PCR,** **QRT-PCR, qPCR, and Sanger sequencing**

| **Primer name** | **Sequence (5’→3’)** | **Locus** | **Usage** | **Product (bp)** |
| --- | --- | --- | --- | --- |
| Dup018_p14F* | ccgagaccaacacacaaatg | NC_000013 114,018,980-999 |  |  |
| Dup018_p14R | gaccgagaagaagccgttc | NC_000013 114,019,123-905 | qPCR | 144 (with Dup018_p14F) |
| Dup023_p57F | ttaaacggtgaggagggatg | NC_000013 114,023,386-405 |  |  |
| Dup023_p57R | catggcctgagcttgttga | NC_000013 114,023,469-451 | qPCR | 84 (with Dup023_p57F) |
| Dup025_p23F | tcttctcccttccccagtg | NC_000013 114,025,567-585 |  |  |
| Dup025_p23R | gagaatgaattcccaccatcc | NC_000013 114,025,677-657 | qPCR | 111 (with Dup025_p23F) |
| Dup041_p17F | tgatgtgtttccaggatctgtc | NC_000013 114,041,257-278 |  |  |
| Dup041_p17R | ttgtcattaccacaccaccac | NC_000013 114,041,333-313 | qPCR | 77 (with Dup041_p17F) |
| Dup228_p72F | ggcaaaacaatggagacagtt | NC_000013 114,228,694-714 |  |  |
| Dup228_p72R | tgcactgccctaaaaatcttc | NC_000013 114,228,793-773 | qPCR | 100 (with Dup228_p72F) |
| Dup237_p13F | gcaaagtgggctgtcatgta | NC_000013 114,237,214-233 |  |  |
| Dup237_p13R | aggacttgaggagctgacca | NC_000013 114,237,282-263 | qPCR | 69 (with Dup237_p13F) |
| Dup241_p17F | cgcaagccatgttcattaca | NC_000013 114,241,291-310 |  |  |
| Dup241_p17R | accagaagcgtgctctaacc | NC_000013 114,241,364-345 | qPCR | 74 (with Dup241_p17F) |
| Dup249_p59F | ctcctgggttgtttgcagtt | NC_000013 114,249,685-704 |  |  |
| Dup249_p59R | ccttgctcattccagacacc | NC_000013 114,249,762-743 | qPCR | 78 (with Dup249_p59F) |
| Dup257_p9F | tcacattcgtagaccctgtca | NC_000013 114,257,807-827 |  |  |
| Dup257_p9R | ccatgctgctagaaaaagctg | NC_000013 114,257,900-880 | qPCR | 94 (with Dup257_p9F) |
| RPPH1_p51F | gcttcggggaggtgagtt | NC_000014 20,811,447-430 |  |  |
| RPPH1_p51R | gccgtgagtctgttccaag | NC_000014 20,811,313-331 | qPCR | 135 (with RPPH1_p51F) |
| ADPRHL1_p18F | atagcctatgacgccctcct | NM_138430 922- 941 |  |  |
| ADPRHL1_p18R | gtacagcaacccgaacagg | NM_138430 1047-1029 | QRT-PCR | 126 (with ADPRHL1_p18F) |
| ADPRHL1_p49F | ctggcacacaccgttcaat | NM_138430 441- 459 |  |  |
| ADPRHL1_p49R | cgctcaggcttccagtacc | NM_138430 530- 512 | QRT-PCR | 90 (with ADPRHL1_p49F) |
| DCUN1D2_p15F | gatggcatgacagaacttgg | NM_001014283 409-428 |  |  |
| DCUN1D2_p15R | ttaaacttggctgtgtccttca | NM_001014283 503-482 | QRT-PCR | 95 (with DCUN1D2_p15F) |

| **Primer name** | **Sequence (5’→3’)** | **Locus** | **Usage** | **Product (bp)** |
| --- | --- | --- | --- | --- |
| DCUN1D2_p78F | ggaacgctgtggacaagaa | NM_001014283 209-227 |  |  |
| DCUN1D2_p78R | aattttgttttcatcttgtggatct | NM_001014283 282-258 | QRT-PCR | 74 (with DCUN1D2_p78F) |
| TMCO3_p10F | cgtggtcaccgaggagat | NM_017905 1975-1992 |  |  |
| TMCO3_p10R | tcgtacgccacaaacgtg | NM_017905 2085-2068 | QRT-PCR | 111 (with TMCO3_p10F) |
| TMCO3_p56F | cacgtgcagatcaccacttta | NM_017905 1152-1172 |  |  |
| TMCO3_p56R | tgtaggcaatcctatggctgt | NM_017905 1246-1226 | QRT-PCR | 95 (with TMCO3_p56F) |
| TFDP1_p61F | gaagcagctcttgccaaaaa | NM_007111 341-360 |  |  |
| TFDP1_p61R | ggtctctgaggcgtaccaatta | NM_007111 421-400 | QRT-PCR | 81 (with TFDP1_p61F) |
| TFDP1_p82F | ctcggctcaggaatgtcag | NM_007111 803-821 |  |  |
| TFDP1_p82R | ttcttgaaggcaatttgctg | NM_007111 916-897 | QRT-PCR | 114 (with TFDP1_p82F) |
| TFDP1_EaF | ctcggccaggaaaaatcat | NR_026580 34- 52 | QRT-PCR | 72 (with TFDP1_EaR) |
| TFDP1_EbF | cgacgctcggccaggaaa | NR_026580 29- 46 | QRT-PCR | 77 (with TFDP1_EaR) |
| TFDP1_EaR | taccagatccgggaaatcaatg | NR_026580 105- 84 |  |  |
| GRTP1_p19F | cagaatcccggctactacca | NM_024719 365-384 |  |  |
| GRTP1_p19R | aggtccggttcaggtctgt | NM_024719 452-434 | QRT-PCR | 88 (with GRTP1_p19F) |
| GRTP1_p66F | agctacctggtcacgctcac | NM_024719 197-216 |  |  |
| GRTP1_p66R | cggacatagcgcttcactg | NM_024719 294-276 | QRT-PCR | 98 (with GRTP1_p66F) |
| Dup275_F | tcggggagttcttctttcct | NC_000013 114,275,560-579 |  |  |
| Dup276_R | cgctagaccacagtccacct | NC_000013 114,276,055-036 | Sequencing | 496 (with Dup275_F) |
| Dup238_F | ccggacacacacagtcagag | NC_000013 114,238,718-737 | Sequencing |  |
| Dup020_R | ggggagggtgaattctgagt | NC_000013 114,020,812-793 | Sequencing |  |
| SGCE_E10F | taatgtagcctagtggccac | NC_000007 94,227,412-393 |  |  |
| SGCE_E10R | agccaacttcatgacttctag | NC_000007 94,226,965-985 | Sequencing | 448 (with SGCE_E10F) |
| rs799917_F | aaccacagtcgggaaacaag | NC_000017 41,245,036-017 |  |  |
| rs799917_R | caggaaagcctgcagtgata | NC_000017 41,244,757-776 | Sequencing | 280 (with rs799917_F) |
| rs861539_F | aagaaggtccccgtactgct | NC_000014 104,165,889-870 |  |  |
| rs861539_R | ccgcatcctggctaaaaata | NC_000014 104,165,646-665 | Sequencing | 244 (with rs861539_F) |

*…continued*

*The number after p is the number in Roche’s universal probe library.
