# Supplementary material for: Dystonia, facial dysmorphism, intellectual disability and breast cancer associated with a chromosome 13q34 duplication and overexpression of TFDP1: case report
Source: BMC Med Genet. 2013 Jul 13;14:70. doi: 10.1186/1471-2350-14-70 (PMC3722009; doi:10.1186/1471-2350-14-70)
Supplement: Additional file 2 — Major genes associated with breast cancer. [file 1471-2350-14-70-S2.docx]

| **Gene**  **(Loci MIM)** | **Cytogenic Location** | **Genomic Coordinates** | **Phenotypes** | **Variants Identified** | **Disease Causing** |
| --- | --- | --- | --- | --- | --- |
| *AKT1* (164730) | 14q32.33 | 14:105,235,685 - 105,262,079 | Breast cancer, somatic (MIM 114480) | *None* | *No* |
| *ATM* (607585) | 11q22.3 | 11:108,093,558 - 108,239,825 | Breast cancer, susceptibility to (MIM 114480) | *rs659243* | *No* |
| *BARD1* (601593) | 2q35 | 2:215,593,261 - 215,674,427 | Breast cancer, susceptibility to (MIM 114480) | *rs1129804*  *rs17489363* | *No*  *No* |
| *BRCA1* (113705) | 17q21.31 | 17:41,196,311 - 41,277,499 | Breast-ovarian cancer, familial,1 (MIM 604370) | **rs799917** | **Unlikely** |
| *BRCA2* (600185) | 13q13.1 | 13:32,889,616 - 32,973,808 | Breast cancer, male, susceptibility to (MIM 114480) | *rs15869* | *No* |
|  |  |  | Breast-ovarian cancer, familial, 2 (MIM 612555) |  |  |
| *BRIP1*(605882) | 17q23.2 | 17:59,756,546 - 59,940,919 | Breast cancer, early-onset (MIM 114480) | *rs4986764* | *No* |
| *CASP8* (601763) | 2q33.1 | 2:202,098,165 - 202,152,433 | Breast cancer, protection against (MIM 114480) | *rs3769823* | *No* |
| *CDH1* (192090) | 16q22.1 | 16:68,771,194 - 68,869,443 | Breast cancer, lobular (MIM 114480) | *rs3743674* | *No* |
| *CHEK2* (604373) | 22q12.1 | 22:29,083,730 - 29,137,821 | Breast cancer, susceptibility to (MIM 114480) | *rs137926355* | *No* |
| *HMMR* (600936) | 5q34 | 5:162,887,516 - 162,918,950 | Breast cancer, susceptibility to (MIM 114480) | *rs34815524* | *No* |
| *KRAS* (190070) | 12p12.1 | 12:25,358,179 - 25,403,853 | Breast cancer, somatic (MIM 114480) | *None* | *No* |
| *NQO2* (160998) | 6p25.2 | 6:3,000,066 - 3,019,993 | Breast cancer susceptibility (MIM 114480) | *None* | *No* |
| *PALB2* (610355) | 16p12.2 | 16:23,614,482 - 23,652,677 | Breast cancer, susceptibility to (MIM 114480) | *None* | *No* |
| *PHB* (176705) | 17q21.33 | 17:47,481,419 - 47,492,241 | Breast cancer, susceptibility to (MIM 114480) | *rs2277637* | *No* |
| *PIK3CA* (171834) | 3q26.32 | 3:178,866,310 - 178,952,499 | Breast cancer, somatic (MIM 114480) | *None* | *No* |
| *PPM1D* (605100) | 17q23.2 | 17:58,677,543 - 58,743,640 | Breast cancer (MIM 114480) | *None* | *No* |
| *RAD51A* (179617) | 15q15.1 | 15:40,987,326 - 41,024,355 | Breast cancer, susceptibility to (MIM 114480) | *None* | *No* |
| *RAD54L* (603615) | 1p34.1 | 1:46,713,366 - 46,744,144 | Breast cancer, invasive ductal (MIM 114480) | *None* | *No* |
| *RB1CC1* (606837) | 8q11.23 | 8:53,535,017 - 53,627,025 | Breast cancer, somatic (MIM 114480) | *rs17337252* | *No* |
| *SLC22A1L*(602631) | 11p15.4 | 11:2,920,950 - 2,946,475 | Breast cancer, somatic (MIM 114480) | *rs1048046* | *No* |
| *STK11* (602216) | 19p13.3 | 19:1,205,797 - 1,228,433 | Peutz-Jeghers syndrome (MIM 175200) | *rs2075607* | *No* |
| *TP53* (191170) | 17p13.1 | 17:7,571,719 - 7,590,867 | Breast cancer (MIM 114480) | *rs1042522* | *No* |
| *TSG101* (601387) | 11p15.1 | 11:18,501,857 - 18,548,502 | Breast cancer, somatic (MIM 114480) | *None* | *No* |
| *XRCC3* (600675) | 14q32.33 | 14:104,163,953 - 104,181,822 | Breast cancer, susceptibility to (MIM 114480) | ***rs861539*** | ***Unlikely*** |

**Major genes associated with breast cancer**

**Dystonia-associated genes**

| **HUGO/OMIM** | **Common Name** | **Locus (Gene)** | **Variants**  **Identified** | **Disease Causing** |
| --- | --- | --- | --- | --- |
| DYT1 (MIM 128100) | Oppenheim's dystonia | 9q34.11 (*TOR1A*) | *None* | *NA* |
| DYT2 (MIM 224500) | Autosomal recessive dystonia | Unknown | *NA* | *NA* |
| DYT3 (MIM 314250) | Lubag (X-linked dystonia parkinsonism) | Xq13.1 (*TAF1*) | *None* | *NA* |
| DYT4 (MIM 128101) | Australian whispering dysphonia family | 19p13.3 (*TUBB4A*) | *None* | *NA* |
| DYT5a (MIM 128230) | dopa-responsive dystonia | 14q22.2 (*GCH1*) | *None* | *NA* |
| DYT5b | dopa-responsive dystonia | 11p.15.5 (*TH*) | *None* | *NA* |
| DYT5b | dopa-responsive dystonia | 2q13.2 (*SPR*) | *None* | *NA* |
| DYT6 (MIM 602629) | mixed-type dystonia | 8p11.21 (*THAP1*) | *None* | *NA* |
| DYT7 (MIM 602124) | familial torticollis | 18p | *NA* | *NA* |
| DYT8 (MIM 118800) | paroxysmal nonkinesigenic dyskinesia (PNKD) | 2q35 (*PNKD*) | *None* | *NA* |
| DYT9 (MIM 601042) | paroxysmal choreoathetosis/spasticity | 1p34.2 (*SLC2A1*) | *None* | *NA* |
| DYT10 (MIM 128200) | paroxysmal kinesigenic dyskinesia (PKD) | 16p11.2 (*PRRT2*) | *None* | *NA* |
| DYT11 (MIM 159900) | myoclonus-dystonia syndrome | 7q21.3 (*SGCE*) | **c.1295G>A**  (NM_001099401) | ***Unlikely*** |
| DYT12 (MIM 128235) | rapid-onset dystonia-Parkinsonism | 19q13.2 (*ATP1A3*) | *None* | *NA* |
| DYT13 (MIM 607671) | Italian family-primary torsion dystonia | 1p36.32-p36.13 | *None* | *NA* |
| DYT15 (MIM 607488) | myoclonus dystonia, Canadian family | 18p11 | *NA* | *NA* |
| DYT16 (MIM 612067) | young-onset dystonia-Parkinsonism | 2q31.2 (*PRKRA*) | *None* | *NA* |
| DYT17 (MIM 612406) | generalized dystonia with dysarthria and dysphonia | 20p11.2-q13.12 | *NA* | *NA* |
| DYT18 (MIM 61216) | paroxysmal exertional dyskinesia (PED) | 1p34.2 (*SLC2A1*) | *None* | *NA* |
| DYT19 (MIM 611031) | paroxysmal kinesigenic dyskinesia (PKD) | 16q13-q22.1 | *NA* | *NA* |
| DYT20 (MIM 611147) | paroxysmal non-kinesigenic dyskinesia 2 (PNKD2) | 2q31 | *NA* | *NA* |
| DYT21 (MIM 614588) | adult-onset mixed dystonia | 2q14.3 - q21.3 | *NA* | *NA* |
| DYT23 (MIM 614860) | adult-onset primary cervical dystonia | 9q34.11 (*CIZ1*) | rs11549266 | *No* |
| DYT24 (MIM 615034) | Dystonia 24 | 11p14.2 (*ANO3*) | *None* | *NA* |
| DYT25 (MIM 615073) | Dystonia 25 | 18p11.21 (*GNAL*) | *None* | *NA* |

NA, not applicable
